# Meaning in Music Is Intentional, but in Soundscape It Is Not—A Naturalistic Approach to the Qualia of Sounds

**DOI:** 10.3390/ijerph20010269

**Published:** 2022-12-24

**Authors:** David Welch, Mark Reybrouck, Piotr Podlipniak

**Affiliations:** 1 Audiology Section, School of Population Health, University of Auckland, Auckland 2011, New Zealand; 2 Faculty of Arts, University of Leuven, 3000 Leuven, Belgium; 3 Department of Art History, Musicology and Theater Studies, IPEM Institute for Psychoacoustics and Electronic Music, 9000 Ghent, Belgium; 4 Institute of Musicology, Adam Mickiewicz University in Poznań, 61-712 Poznan, Poland

**Keywords:** music, soundscape, sound environment, soundscape descriptors, musical affordances, coping behavior

## Abstract

The sound environment and music intersect in several ways and the same holds true for the soundscape and our internal response to listening to music. Music may be part of a sound environment or take on some aspects of environmental sound, and therefore some of the soundscape response may be experienced alongside the response to the music. At a deeper level, coping with music, spoken language, and the sound environment may all have influenced our evolution, and the cognitive-emotional structures and responses evoked by all three sources of acoustic information may be, to some extent, the same. This paper distinguishes and defines the extent of our understanding about the interplay of external sound and our internal response to it in both musical and real-world environments. It takes a naturalistic approach to music/sound and music-listening/soundscapes to describe in objective terms some mechanisms of sense-making and interactions with the sounds. It starts from a definition of sound as vibrational and transferable energy that impinges on our body and our senses, with a dynamic tension between lower-level coping mechanisms and higher-level affective and cognitive functioning. In this way, we establish both commonalities and differences between musical responses and soundscapes. Future research will allow this understanding to grow and be refined further.

## 1. Introduction

Sound has a sound: the external, physical/acoustic vibrations that are called sound create within our minds perceptual phenomena that we also call sound. Is there a distinction between sound, in either of these senses, and music? Should we conceive of sound and music as orthogonal categories in a statistical sense, or should we instead think of a continuity and a gradual transition between them? The latter implies that sound should be the more inclusive category with music being a kind of subcategory with special characteristics and constraints, which relate to the use of preferred frequencies and harmonics that were selected through centuries of refinement and development of music as a distinct category [1,2].

These questions form the backbone of this contribution. It tries to bring together existing approaches and definitions, related to sound and soundscape, in an attempt to provide a provisional theoretical framework that revolves around existing definitions and descriptions of many related terms and the methodological challenges to assess them. It starts with a naturalistic description of sound and music to elaborate further on the possibility of structuring and organizing sound so as to make sense of it. It then critically examines the construct validity of the term soundscape and expands further on the qualia of sound environments, and the way listeners cope with their surrounding sonic world.

At an acoustical level, first, music is different from all other sounds, due to the complex vibrations produced by musical instruments and singing voices. This holds for the distinction between musical sounds and the distinct sounds of nature, but the distinction is still more dramatic with respect to those sounds that are categorized as noise (according to one of its definitions), and which are characterized by inharmonic sound vibrations and irregular frequency relations between the constituting vibrations (see [3]). Neonates, for instance, are capable of discriminating immediately between music and ambient noise by recognizing that music as an auditory structure is qualitatively different from the disorganized noise that surrounds them [4]. They seem to show a preference for the aesthetic coherence and organizational structure of music with the ability to pick out fine-grained formal properties of music, such as pitch, melody, tempo, and musical phrase structuring [5,6,7]. In the same vein it should be mentioned that not only newborns but even skilled musicians who claim to like dissonant music exhibit enhanced electrodermal activity in response to dissonant music as compared to consonant versions of the same piece [8]. There seem to be reflex-like responses to either dissonance or consonance with underlying mechanisms such as the roughness created at the level of the basilar membrane in the inner ear in the case of dissonance [9] or the opportunity for consonance calibration at multiple levels along the auditory pathways. Consonance, moreover, is ubiquitous in the auditory environment in the sense that most natural sounds, and speech in particular, consist of consonant harmonic intervals. They appeal to central neural networks that are preferentially attuned to consonant intervals as the result of a process of generalization because of their prevalence or significance in the sonic environment [10,11,12].

It can be asked, further, whether these characteristics and constraints relate to the acoustical properties of the music or to the listener. For example, although the acoustic waves produced by human voice during speaking and singing differ (among others) in terms of F_0_ stability [13] the repeated spoken phrases can be heard as song [14,15,16]. The same can be true for the repetitions of environmental sounds experienced as music [17], which suggests an important role of cognition in the process of sound interpretation as music. Moreover, it is possible, in fact, to “musicalize” all possible sounds by giving them some music-semantic weight. Yet, some sounds lend themselves more easily to some musical designation than others (see above), and there are actually acoustic descriptors which can be used to assign some musical value to selected sounds [1]. However, in most cases, music can be seen as a collection of acoustic events which are organized in some way. It allowed avant-garde composers such as Edgard Varèse and John Cage to go beyond classical connotations of the term music to open up new modes of composition by referring to their music as “organized sound” or “organization of sound” [18]. It can be questioned, further, whether this organization is part of the intrinsic structure of the acoustic events, or whether it is imposed on them by the mind of the listener [19]. However, broadening the scope of music to include all kinds of sounds has the advantage of drawing attention to the exploratory and interactive nature of listening to the “sonic world” in the broadest sense. This entails the possibility of experiencing the “world-making” power of music, in the sense that organized sounds can be used as a tool for creating, organizing, and regulating our experiences and our relationship to the world [2].

In this paper we take a naturalistic approach to music and music listening, in an attempt to describe in objective terms some mechanisms of musical sense-making and interactions with the sounds. Rather than engaging in sterile discussions about the music/non-music distinction, we take as a starting point a definition of music as vibrational and transferable energy that impinges on our body and our senses, with a dynamic tension between lower-level reactive processes that rely on older evolutionary levels of coping with sounds and higher-level affective and cognitive functioning. There is, as such, an interesting complementarity of bottom-up and top-down processing of the sounds [20].

## 2. The Soundscape and the Response to Music

While both the soundscape and the response to music have parallels, research into describing the two types of experience has not yet overlapped. Here, we describe the basic research in these two areas.

### 2.1. Conceptualising the Soundscape

The concept of soundscape has a rather short history, which means that it may still have flaws and weaknesses. It has a background in acoustic ecology [21,22], but research within the field of musicology is rare and to some extent still lacking. There are some contributions from ecomusicology—a discipline at the intersection of music, sound, culture, society, nature and environment [23,24]—and ethnomusicological research [25].

As is the case in new emerging fields in science, there is, at first, an immature stage that is characterized by disagreement on principles, methods and even accepted facts. It means that scholars still have to come to agreement on a unifying paradigm to guide their research [26]. There are, as such, many related terms that are used interchangeably without always providing clear and valid definitions. This holds, first of all, for the concept of soundscape, which is closely related to the acoustic environment and the way this is perceived. Within this construct, a distinction must be made between soundscape design, soundscape descriptors, and soundscape appraisal, with a dynamic tension between objective, acoustic descriptions and subjective evaluations of these environments [27,28,29,30,31,32,33,34].

The concept started gaining traction after the establishment of The World Soundscape Project by Schafer during the late 1960s and early 1970s as the outgrowth of his initial attempt to draw attention to the rapidly increasing noise pollution of the acoustic environment [35,36] and follow-up studies by Truax. It was an approach that gave impetus for soundscape ecology, as an umbrella term for landscape ecology and acoustic ecology [37,38] as a logical prolongation of Schafer’s soundscape studies. In an effort to propose a positive alternative to previous negative, anti-noise, approaches, he proposed a listener-based approach relying on technique of ‘‘ear cleaning’’ and ‘‘soundwalks’’ to counter the negative effects of soundscapes that produced a habituated response of non-listening to the acoustic environment.

The term soundscape has been described by Truax as “an environment of sound (sonic environment) with emphasis on the way it is perceived by a person or people, or by a society” [38] (p. 126) (and see also [33]). Since those days, the term soundscape is defined in two main ways: one, as defined by the International Standards Organisation in ISO12913 is the “acoustic environment as perceived or experienced and/or understood by a person or people, in context” [39]; the other is as a synonym for the acoustic environment. We will adopt the former usage, so consider the soundscape to be a perceptual phenomenon, that is influenced by the sound environment, but also by other sensory information, memories, and states and traits of the person in which it manifests.

The sound environment should be seen as a necessary and influential precondition for the soundscape, and has been considered in detailed research in the context of *auditory scene analysis* and the related search for auditory streams in the environment [40,41,42]. This field addresses the problem of how listeners can hear in complex auditory environments by integrating findings from psychoacoustics, speech perception, music theory and composition, and computer modelling. A major challenge in this regard is to distinguish between the massive overlap of meaningful acoustic signals and the sounds from the wider surroundings. It is still a matter of debate whether the former may trigger our attention in a quasi-automatic way or whether they are the outcome of a listener’s focus of attention, though there is some agreement about the attention-capturing potential of some stereotypic sounds [43].

Schafer’s original distinction between *hi-fi* and *lo-fi soundscapes* can be considered as an interesting starting point in this regard. Starting from the signal to noise ratio, he conceived of a hi-fi system as one in which “discrete sounds can be heard clearly because of the low ambient noise level”, while “in a lo-fi soundscape individual acoustic signals are obscured in an overdense population of sounds” [44] (p. 32). The countryside is more hi-fi than the city, and ancient times were also more hi-fi than modern times. The distinction, however, has consequences for the processing of information, and, above all, for the processing efforts, in the sense that in a hi-fi soundscape even the slightest disturbance can communicate interesting and vital information. It allows the ear to function as a sentinel, celebrating its primary alerting and motivational role in preferring certain environments and avoiding others. Hi-fi soundscapes, therefore, should be favorable for survival purposes since they make the signals easier to process, thus reducing the complexity of their analysis (see also [33,45]). In lo-fi soundscapes, on the other hand, individual acoustic signals are mainly obscured by the masking effect of a population of sounds. Everything is “close-miked” with cross-talk on all the channels, with the resulting need of amplification of even the most ordinary sounds to be heard. It is clear that this has consequences for the ecology of listening [3].

The complexity of soundscapes refers to the number of competing auditory streams in a larger search space [40] and the related difficulty to process its available affordance content in terms of appropriate behavior [46]. It brings us to the second part of the definition, namely the decision-making process of meaning attribution and appraisal of the environment. This seems to be determined to a great extent by the degree of subjective control (see [33] for an overview).

There have been several attempts to describe how people perceive the acoustic environment, defined in operational terms as soundscape descriptors, which provide a dimensional structure of soundscape indicators, and which reflect meaning attribution rather than merely describing the physical characteristics of the sound [27,31]. These descriptors have been generated in a variety of ways and from different theoretical backgrounds, which is a likely explanation for the range of different types. One class of soundscape descriptors has arisen from questionnaires that are restricted to affective aspects of the soundscape, e.g., [47], and have a theoretical basis in Russell’s work on the two dimensions of affect [48]. In this work, dimensions mirroring Russell’s two dimensions, Pleasantness (emotional valence) and Eventfulness (vibrancy) have been identified [31,49]. A calm environment affords indications of safety and allows people to restore resources; a lively environment is stimulating and safe and makes it possible to learn and play; a boring environment does not guarantee a sense of safety and control; and a chaotic environment contains indications of insecurity and danger [33]. Other related work has preserved the pleasantness/eventfulness dimensionality and added a third component: “Familiarity” [28], the sense that a soundscape is known; “Restorativeness,” the sense that a soundscape helps people to recover from tiredness or malaise [50], or “Appropriateness,” a sense that the soundscape is right for the place in which it is experienced [46].

Other research has included qualia of the sound environment in addition to the affective aspects. Originally, qualia referred to the intrinsic qualities of a subjective experience that is associated with a given sensory object [51]. In more recent research, the term has been used to describe subjective experience more broadly [52] and also in musical contexts [53]. It may be that, in the context of listening to sound, people do not distinguish their emotive responses to the sound from the experience of the sound itself: the qualia.

This has added greater dimensionality to the picture. For example, one model has produced descriptors: “Relaxation”, “Communication”, “Spatiality,” and “Dynamics” [54]. Interestingly, in these models, people do not separate the affective aspects of the soundscape cleanly from the qualia. For example, one model provided: “Uplifting” as a purely affective component, “Hectic” and “Stable” as purely related to the qualia of the sounds, and “Demanding”, which combines the influences on both affect and qualia [55]. This may imply that people do not separate their emotional response to a sound from the experience of hearing the sound.

### 2.2. Conceptualising the Musical Experience

Like the soundscape, different models have been developed to attempt to explain how we perceive music and how it influences us emotionally [56,57]. As with attempts to model the soundscape, research into the response to music has been based on theories of emotion. In particular, Russell’s model, capturing the two dimensions of emotional valence and arousal has been influential [48], though the application of the model to musical experiences anticipated Russell’s work by over four decades [58]. A model of the emotional response to music that is unlike any as yet proposed for the soundscape is a hybrid model [56], which captures three levels of emotional response to music: low-level, core affect; phylogenetically older basic emotions; and high-level phylogenetically younger emotions such as nostalgia or awe, which are not captured by the simpler emotional states of the lower levels. On the other hand, this division of emotions according to levels can be contrasted with a functional perspective in which each emotion evolved separately and because of different functions [59]. 

Another important aspect of the response to music which has received little attention in the soundscape field was also anticipated by Hevner, when she referred to music as a “temporal art” [58] (p. 201), emphasizing the ever-changing nature of music, and that it is changes (e.g., in pitch or rhythm) within a piece of music that leads to changes in affect in a listener. More recently, the inability of theories of emotion to capture the ever-changing and complex flow of emotions that humans experience has been commented upon by McCrae [60], raising the intriguing idea of the potential for music to illustrate or capture those changes. In recent research investigating the soundscape using a questionnaire followed-up by interviews, participants referred to the difficulty of expressing their experience of an ever-changing soundscape using a questionnaire completed at a single point in time [61]. Perhaps a truer approach than a static questionnaire to describe soundscapes may be through a form of musical composition, as suggested by McCrae, that allows a listener to extract and present the salient aspects of the soundscape through a more dynamic medium.

## 3. Music as Soundscape

It can be questioned whether there is a difference between sound and music [19]. A possible answer is to consider the semantics of music, and the process through which listeners apply their knowledge of the conventions and understanding of music to the sounds they hear when they are listening to music as opposed to other types of sound. Here, we look to extend this thinking by comparing music to soundscapes. Soundscapes too can influence people in part through the knowledge of the world of which the soundscape is a reflection. As with the perception of music, the human response to soundscapes has evolved and is intrinsic to our auditory nervous systems. Since music may also have influenced the evolution of the same structures that mediate the soundscape, can music be regarded as a kind of soundscape? It may be that experience of music (musical literacy) can influence our experience of soundscapes [41].

### 3.1. Soundscapes and Their Qualia

The perception of music certainly has an affective influence on listeners, and the qualia of the sounds of the instruments and/or recorded music are also apparent to a listener and they interact with the affective aspects of the experience in important ways. Most soundscape research has focused on affect, but it has been shown that both affect and qualia of the sound environment interact in our experience of the soundscape [55,61,62,63]; this may suggest some commonality between musical percepts and soundscapes, but are they really the same?

Truax has suggested that speech, music, and soundscapes can all been regarded in a similar way [64]: in his view, each of them depends on some external acoustic signal but is interpreted and given richness by the internal, perceptual processes that define our experience of each. He makes the point that proper understanding of the perceptual aspect of sound relies on much more than the acoustical quantities of the sounds, but while it begins with those, it also involves cultural, anthropological, historical, and psychological contexts that give the context important for understanding when listening.

In this context, the possible gene-culture coevolution processes that could have shaped human musicality [65,66,67,68] and speech abilities [69,70,71,72,73] should also be taken into account. According to all these co-evolutionary scenarios, the crucial role of music and speech in our ancestral prehistory has become a part of the selective environment leading to the evolution of species-specific perceptive abilities in the domain of audition.

We have previously defined music as “a sound environment, which encompasses both natural and man-made sounds” [20] (p. 2). Both soundscapes and music are associated with affective responses, and both include qualia, the perceptual representation of the acoustical qualities in the environment. Clearly there is a link between them, but what is it, how do they differ, and in what ways can one be part of the other? Music can occur in soundscapes when the sounds that make it up are part of a greater sound environment. On the other hand, can the perception of a piece of music be regarded as a soundscape in itself: or is listening to music, as a phenomenon, different from experiencing a soundscape?

For the answers to these questions the distinction between music and sound art [74] or music as an art that is based on a human-specific form of sound communication and music as an art which lacks this human-specific form of communication [75,76] seems to be useful. Music based on this human-specific form of communication is perceived as a kind of Humboldt system [77] that consists of spectro-temporal patterns of discrete pitches and rhythm measures. Music that lacks these patterns is composed of sounds, e.g., natural or industrial sounds, which can be interpreted as music only if we are aware of their intentional “musical” use as in the case of *musique concrète*. From this perspective, the perception of the former differs from the perception of the latter in terms of cognitive processing. While the auditory scene analysis [40] is an indispensable perceptive strategy that is present in both types of music, only the perception of music which is based on a human-specific form of sound communication involves certain additional cognitive abilities. In both cases, however, the awareness of sound patterns is crucial for their aesthetic experience. Since soundscape can be also a subject of aesthetic appreciation and music can be perceived without its awareness, the question about the difference between music and soundscape still remains open to some extent.

### 3.2. Music in a Soundscape vs. Music as a Soundscape

Most soundscape research has focused on the soundscape as a response to acoustical environments that occur without deliberate intervention. In such research, manmade sounds are often present, but typically as by-products of other activities (e.g., traffic or construction machinery), or the sound of people (e.g., footsteps or speech), while natural sounds (e.g., birdsong or running water) are the main environmental elements considered. The question of music as a soundscape has generally not been addressed. Since most soundscape research has focused on the sounds that occur naturally (e.g., [78]) they might well include music, for example, music from a passing car in the street, but it is not music that is central to the sound environments. In some applied research, music has been used as part of a broader sound environment aimed at creating positive soundscapes, for example in a healthcare setting [79], but whether the music creates a soundscape was not questioned; rather, the music was seen to serve a role in the overall acoustic world that was created. In another example, music was shown to increase the length of time people spent in a public place [80].

In a project where people could play their own music from phones and music players through speakers housed in a gazebo in a public park, music was introduced to the sound environment of the park and thereby caused positive changes to ratings of the soundscape [81]. In this case, the park was small and near a busy road, while the musical sounds were contained to some extent by the location within the park, so the music provided only a part of the total soundscape for most park users. Another example is the use of music in neonatal intensive care units. When mothers sing to their babies this influences the soundscape positively by humanizing it and demonstrating affection [82]. In general, music appears to lead to health benefits, and the mechanisms underlying this are likely to be the same as for soundscapes [83].

The above examples are of music being part of a typical soundscape: music in a soundscape. To consider a piece of music as a soundscape is more challenging. If a soundscape is the perception of an acoustic environment by a person, and if the music dominates the environment, as an orchestra does in a concert hall, or a pop song played via earbuds does wherever you may be, then the environment is entirely music, and so, by definition, it creates the soundscape. Listening to music provides a different kind of experience than listening to a typical acoustical environment though. Music deliberately delivers a message to an audience, whereas the message in a natural soundscape, though present, is rarely deliberate.

Truax, through his involvement in electroacoustical music, regards music as a soundscape [64]. This might be a special case, where the electronic manipulations to the musical sounds are used to convey soundscape-like percepts to listeners. Such electroacoustic music uses and manipulates the electrical signals generated by or recorded from musical instruments. Some of the manipulations can mimic the acoustical changes that occur when in a natural sound environment. For example, part of a piece of music might include changes to the localization of the sound sources due to artificial alterations in the phase/level of the sound [84]. However, what about more conventional styles of music?

Some music contains environmental elements, and listening to that music would evoke aspects of a natural soundscape. In our previous writing, we have considered music through the same lens as the soundscape [20]. It is a conception that argues for a continuity between the perception of natural environments and music, as advocated by Dewey, who defined a real experience as heightened vitality, signifying active and alert commerce with the world, which, at its height, signifies complete interpenetration of self and the world of objects and events [85] (p. 19). Even is there may be a qualitative distinction between music and natural sounds, the way of listening appeals to the same mechanisms of exploratory behavior and focused attention. It makes sense, therefore, to broaden the scope of music listening to embrace also the biological underpinnings of sensemaking of the environmental world in general, and to conceive of music not only as an aesthetic artefact, but also as a vibrational phenomenon that can be considered as a human-made soundscape that impinges on our body and our senses in the same way as natural soundscapes do.

### 3.3. Soundscapes in Music

It has been proposed that there are musical elements in naturally occurring acoustical environments [86]. Soundscapes include qualia and affect, as does music. When we listen to music, we feel emotions and experience the sounds of the instruments, but we also follow a development that occurs over time. This kind of development might be observed in a soundwalk, where a listener experiences the changing soundscape of a route, for example through a city [78].

The *soundwalk analogy* can be applied also to listening to music. It is possible to conceive of music in general as a soundscape, but it is possible also to conceive of distinct soundscapes in the course of one single piece of music. This is quite obvious in the case of large-scale symphonic classical music with huge orchestras, where the distinct instrumental sections are not only physically grouped together in the spatial arrangement on the stage—as a kind of “acoustic biotopes”—, but where the sounds they produce can be considered also as qualia that determine to some extent the “feel” of a specific acoustic environment. It is an approach that offers interesting perspectives with respect to the development of listening skills.

Let us take an example to clarify a little: Sibelius’ second symphony, first movement. This symphonic work has been conceived for a common instrumentation, namely 2 flutes, 2 oboes, 2 clarinets, 2 bassoons, 4 horns, 3 trumpets, tuba, timpani, and strings, and takes typically the form of an evolution of themes. It starts with fragmentary, inchoate motifs that gradually coalesce into more full-fledged musical ideas. As is clear from the spectrogram, the music is a succession of short acoustic events that are easily distinguishable from each other, each of them being characterized by a specific spectral configuration. There is, as such, a clear distinction between the heroic brass fanfares as against the soft sounds of the woodwinds, the overtone-rich sounds of the strings, and the threatening sounds of the timpani.

**Figure 1:**
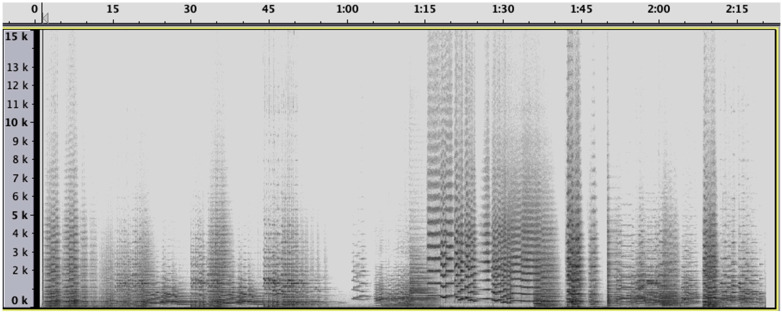
Spectrogram of the first 2 min of Sibelius’ second symphony, first movement. The picture reads as a succession of distinct acoustic biotopes.

The experience of a live performance adds a lot to the recognition of these instrumental qualia, due to their specific location on the stage, with contrasts in the instrumentation being reflected and made visible also in their spatial position as sound sources with their typical place in the orchestra. A spectrogram of the first two minutes is depicted in Figure 1.

**Figure 2:**
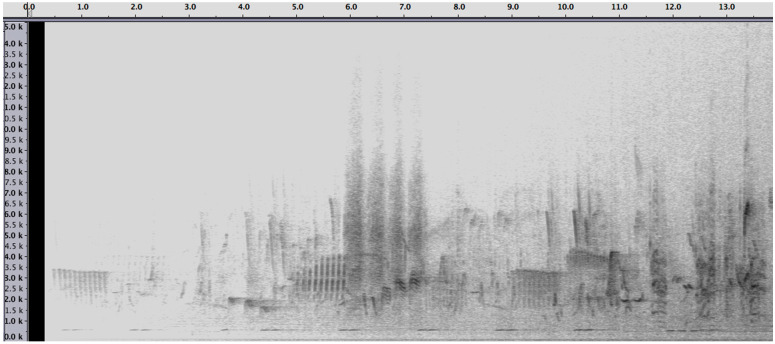
Spectrogram of about 13 s of the acoustic biotope of a typical African rain forest. Most of the sounds are animal sounds with both harmonic and inharmonic constituents.

The specific texture of such musical “biotope” is particularly clear when compared to, e.g., an acoustic snapshot of a typical African rain forest, as depicted in Figure 2. The global picture is more random with lots of both harmonic and inharmonic sounds. Some of them, in particular bird sounds, are quite recognizable and identifiable as such, though this does not yet transform them into music. It is possible, however, to musicalize these sounds, when one takes a certain aesthetic position that locates the aesthetic value in the intention of the listener.

The spectrograms, moreover, raise some challenging questions as to how to interpret them. Even if the global picture may seem to be a patchwork of separate and unrelated entities, it is clear, at first glance, that some regularities emerge with individual elements that can be labeled in terms of distinctions and observables, even if the labels are not yet at the listener’s disposition. It raises also another question of temporal “resolution”: how long must the excerpt be to function as a soundscape? Do we conceive of the whole, global picture as one soundscape, at a kind of macro-level, or can we conceive also of micro-soundscapes, which refer only to some shorter excerpts that are recognizable as such? Additionally, should we conceive of soundscapes as monolithic blocks or is it possible also to conceive of the simultaneous combination of distinct soundscapes in the same acoustic environment, somewhat analogous to the ecological concept of biotopes? To the best of our knowledge, this avenue has not yet been investigated in depth, yet it offers major possibilities for future and extant research.

### 3.4. Insights from the Responses of Other Species

As the naturally occurring terrestrial sound environments, that have accompanied animals’ life since their conquest of land, have been relatively stable [87], humans share with other tetrapods many cognitive mechanisms enabling auditory scene analysis [40]. However, due to different evolutionary trajectories of different phylogenetic lineages, the experience of a natural soundscape by various animals can be also composed of species-specific elements. This is especially important for animals that communicate vocally. For instance, on the one hand, vocalizations of the majority of tetrapods share some “voice modulatory cues” as a result of similar evolutionary pressures [88]. On the other hand, animals’ songs are very diverse and the comprehension of similar signals by different species can differ depending on their songs’ specificity, as in the case of recognition of starling songs by humans and starlings [89]. As speaking and singing are human species-specific forms of vocalizations, one can assume that our language faculty [90] and musicality [91,92,93] influence our experience and interpretation of music and speech sounds in human-specific way.

Even if a natural experience and a musical experience share the same underlying mechanisms of perception, cognition, and appraisal, it can be questioned whether there are any features of the one that are not possible in the other. Soundscapes generally result from naturally occurring sound environments, whereas the response to music is generally a result of sound that is composed. Even if natural elements are included, they are chosen deliberately because of the way they will make a listener feel. The intentionality in musical composition does not necessarily mean that our response to a piece of music would differ from a soundscape occurring in response to a natural sound environment, but our awareness of the forms of music and of the composer’s intentionality may introduce different elements.

Consideration of non-human animals may provide helpful insights to understand this. Each species tends to respond to a given sound environment in different ways, since each species evolved to respond in species-specific ways to aspects of the sound environment relevant to its survival. There is ample evidence, from presenting recordings devoid of any other cues, that animals’ behavior changes in response to environmental sounds. For example, gorillas held in captivity were exposed to recorded rainforest sounds, and while infant gorillas showed more relaxed behavior, adults became more active [94], whereas in a later study, adult gorillas appeared to become (statistically marginally) more relaxed in similar conditions [95]. Interestingly, the later study also exposed the gorillas to randomly changing classical music tracks and found that behavior was even more relaxed than during exposure to the rainforest sounds. This may imply that animals most similar to humans derive some similar experience from music. In other research, monkeys could identify a simple melody as the same even when it was transposed by an octave, despite being capable of discriminating between the frequencies of the transposed tones when presented outside the melody [96]. This may suggest that not only is the response to music similar, but the appreciation of the characteristics may be present.

Animals’ responses to music have also been shown in species less closely related to humans. For example, parrots can exhibit behavior similar to human dancing in that they will move in time to the beat of a piece of music [97]. The authors of this research point out that parrots’ brains are like humans’ in the respect of having strong audio-motor connections, which other species, that do not move to musical rhythms, do not. This idea that musical appreciation must be relevant to the species in question has been applied in developing appropriate “music” for other species. While cats do not appear to respond to music written for humans, they do respond to music designed for cats [98]. Starting from the perspective that human music may evoke emotions in humans via the principle of emotional contagion, and drawing on research showing that the affective qualities in human music parallel those of human voices [99], music was developed for cats that contains sounds similar to purring and other cat vocalizations, while avoiding sounds similar to cat cries associated with apparent negative emotions. They found that cats (especially those under about five years of age) showed more behaviors such as orienting to the speaker, rubbing against the speaker, etc. for the music designed (by humans) for cats, whereas they demonstrated little interest in or awareness of the human music [98].

In general, music can be defined at an acoustical level as organized sound (see [19] for an overview, and though sound environments can also potentially be organized, they lack the level of detailed, semantic, organization of a piece of music. For humans, music is a presentation of ideas by the composer and performers to the listeners. As such, it implies a kind of intentionality. The soundscape, on the other hand, emerges from any sound that a person hears; it may contain meaning, and it is possible that some of the meaningful aspects of the soundscape were deliberately introduced, but that is not usual. Rarely would a soundscape be completely dominated by a single flow of information: perhaps a conversation in a quiet room or a public lecture would be like that. A beach with crashing waves that mask all other sound would also be dominated by one sound: but the waves convey little information, and the structure is very simple compared to the other examples. For animals, which presumably lack awareness of the intentionality in music, there is evidence that they respond to music when it has relevance to them.

All this shows the difficulty of attempts to delineate and demarcate the boundaries of both music and soundscape. It is possible, however, to see not only the distinction, but to focus rather on the commonalities to find out how listeners, be they human or animal, deal with both of them. Through mimicry of elements in the acoustic environment, birdsong may potentially incorporate elements of a bird’s soundscape and, in human speech, onomatopoeia occurs where words sound like their meaning (e.g., “rumble”). This tells us that some aspects of a sound environment are available to humans and animals, and that elements of it may be co-opted and used for effect. Listening, in that broader view, can be conceived as a way of coping with the sonic world.

## 4. Coping with the Sonic World

As mentioned above, the concept of soundscape entails the interaction between a person and his/her environment, thus consolidating informational, affective and activity related perspectives on its appraisal [33]. As such, there is some analogy with the ecological approach to perception, which also stresses the role of interaction of an organism with its environment. Translated to the field of music, this should mean that we conceive of the listener as an organism and of the music as a sounding environment [100]. Perception, in this view, is ecologically constrained, which means that we address the world not in terms of its physical description, but in terms of survival and orientation in the environment [101]. It is a conception that goes beyond the dichotomous approach of organism-environment dualism in favor of an approach that is not animal-neutral and that conceives of the environment as perceived by the (human) animal.

Music listening, in this ecological view, is also not neutral, but is affordance-driven. It means that the sounds may have a demand or invitation character or valence, as coined already by early Gestalt psychologists as Koffka and Lewin, who pointed to the importance of functional and semantic relations that biological organisms establish with their environment [102,103] (and [104,105] for a broad overview). Furthermore, the emotional response may be complex, as determined by the affordances of each environment. Affordances, as the term was defined by Gibson, can be equated with environmental support for an organism’s intentional activities. They must be seen as subjective qualities that render them apt for specific activities—such as supporting locomotion, concealment, manipulation, nutrition, and social interaction of the animal—, which make them meaningful for active perceivers. They thus must be seen as opportunities for goal-directed action, in the sense that something can be perceived in terms of being graspable, climbable, having a surface that can be walked on, sat on, hidden under, picked up, climbed over or thrown. It means also that affordances are specified in relation to the specific anatomy and motor capacity of the animal or organism, which define to some extent the specific action repertoire and skills, which are unique to each individual animal. The sensitivity to different affordances is thus dependent on both the biological disposition and the developmental and experiential history of the animal [2].

As such, affordances refer to both the environment and the organism, not in a kind of dualism but in terms of the complementarity of the organism and its environment [106] (pp. 12 and 127) (see also [107]). The concept can be easily translated to the domain of music if we understand music in terms of what it affords to us and not merely in terms of an acoustic description of the sounds [101,102,103]. Music, in this view, can be perceived as an affordance-laden structure, with musical experience being seen as an exploratory activity with perception, manipulation and appropriation of different sonic affordances, offered by the music. It is important, in this regard, to keep in mind that the concept of affordance should be interpreted in the broadest way, including both emotional and social affordances. It means that joint attention to music alters the way music is perceived in social listening contexts [2].

It is possible, in this vein, to see soundscapes as an analog of musical affordances, in the sense that they represent a kind of meaning attribution of what the acoustic environment offers, provides, or furnishes the individual listener, either for good or ill [33]. There is, however, no causal relation, as listeners have a lot of perceptual autonomy in the way they listen, in the kinds of meaning they attribute and the way they engage with music to forge relationships and shared experience [108].

In the context of coping, the environment can drive an organism towards two different poles of a continuum: at one pole, the problem-oriented reactive *survival or coping mode*, which aims at ending or avoiding threats to existence and protecting viability in a deficient environment; the *flourishing* or *co-creation mode*, at the other pole, occurs when survival is not immediately threatened and aims at improving viability and to create better conditions for living, such as sexual opportunities, food, which increase the chances of reproduction and survival, respectively [109]. The survival mode, which prevails in situations of low viability, is mostly experienced as unpleasant (aversive), whereas the flourishing mode is perceived mainly as enjoyable (attractive), with as underlying mechanism the relation between indicators of safety, affective appraisal of soundscapes and the inner affective state or core affect [33].

The coping mode has received a lot of academic interest since Lorenz’ ethological work on survival value. His description of the animal in action in its natural surrounding has had a strong appeal to naturalists who saw animals having to cope in numerous ways with a hostile or at least uncooperative environment [110,111] (see also [112]). Coping, however, is a broad category. It is defined mainly as a survival mechanism of a living organism in its interaction with the environment, involving both behavioral and cognitive efforts to manage specific external and internal demands [113]. As such, it involves not only reactive behavior, but it involves also processes of attributing sense to the environment ranging from manifest physical reactions over affective-emotional reactions to mental and cognitive operations. This can be applied to our interaction with the environment in general, but also to our interaction with music, defined as a sonic environment. Listening, then, can be defined as coping with the sonic world [20].

Acoustic design can draw upon biological aspects of coping to create improved soundscapes for people. Human-created acoustic environments can be dominated by mechanical sounds, which decrease the perceived tranquility, whereas natural sounds may enhance them [114]. One of the challenges for acoustic design, therefore, is to pay special attention to the “repatriation of quiet grooves and times” and to create acoustic spaces that are quiet and unpunctured by sound [44].

Empirical research has been done on the effects of noisy environments, especially on newborns and premature babies who suddenly are forced to leave the security of the womb for the noisy environment of the neonatal intensive care unit (NICU). The ambient sounds that pervade this environment—consisting of sounds that are random byproducts of medical instruments, such as clicking, beeps, and whirring from respirators, incubators, heart monitors, and others—without control for their volume, duration, location, or cause/effect relationships can have unfortunate consequences, such as fatigue, stress, hyperalerting responses, startle reflex, and many others. They collectively shape a noisy, chaotic and uninviting sound world which disrupts the neonatal biorhythms, adversely affecting sleep regulation and state lability [2,115,116]. There have been concerns in the medical community, therefore, about the harmful effects of these ambient auditory stimuli with recommendations for greatly reduced decibel levels [1].

Studies have recommended the use of soothing music in these units, and in other areas of hospitals [117,118,119]. This work has a strong basis in psychophysiology, and explicitly draws upon some of the principles of the soundscape, as has been well explained by Rossetti [120]. It is also possible to generalize this to the widespread use of music for babies and young children, as is the case with lullabies and playsongs, which are sonically interesting but perceptually quite undemanding. Most of this music is texturally soothing with no abrupt modulations of volume and tempo, and is relatively unchanging with respect to melodic and rhythmic patterns. As such, it is music with thin textures that reduces alerting responses [1].

Such music does not overwhelm the infant and affords a kind of environmental autonomy that secures simultaneously security and stability [47]. It allows the infant to focus and explore the music by actively foregrounding it in the perceptual field, but it can tune out as well, allowing the music to recede in the background. The infant, then, can manipulate to some extent, its sonic environment [2]. Much is to be learnt here from the domain of infant-directed singing with typical acoustic modifications such as a slower tempo, more relative energy at lower frequencies, longer pauses between the phrases, more pitch and jitter factors, more pitch variability and more exaggerated rhythms [121,122,123,124,125,126,127]. In biological terms, this means that these acoustic interactions are presumably among the least aggressive of human interactions. In other words, they are easy to cope with.

## 5. Beneficial Effects of Music Listening: Soundscape Selection and Sonic Design

The way we are dealing with music has evolutionary motivations, which reflect our basic homeostatic functioning in the sense that it should assist in maintaining a state of healthy equilibrium [2,128]. Two main mechanisms are important in this regard: the need of restoration and relaxation on the one hand, and the need of environmental enrichment to provide optimal stimulation on the other hand. The first can be explained in terms of Kaplan’s *Attention Restoration Theory* [129], which states that prolonged periods of directed attention cause attentional fatigue that needs to be recovered in restorative environments. Such environments, as a rule, do not require high processing efforts, due to the lack of complexity and redundancy of easy to process indications. They are mainly tranquil, they leave a harmonic impression, and are rich but without demanding focused attention, and are exemplified most typically in natural environments, which provide a kind of audible safety [50,130]. The second mechanism stresses the role of auditory exposure from birth to provide the critical needed stimulation for further development of our auditory abilities as well as general cognitive development [1]. This makes these sounds interesting, so they attract attention.

Listeners can choose which sonic environments they engage with, but only to some extent. Some of them are part of our natural environment, others are imposed on them by external circumstances or are the outcome of deliberate and selective exploratory behavior.

Natural soundscapes are part of our developmental ontogenetic history. A prototypical example is the stable and predictable intra-uterine environment in the womb, for which the description “uterine symphony” has been coined [131]. It consists of the soothing maternal sounds of breathing, digestion, the heartbeat, and other sounds of the mother’s body [132], which undergoes a dramatic change when the rhythm that the fetus has become accustomed to through months, is replaced by the much harsher sounds of extrauterine life. It is one of the most stressful changes that the infant faces during the transition from intra-uterine to extra-uterine life. One of the answers to this threat is the widespread use of *lullabies*. They have been sung in all cultures to afford security and emotional warmth [124], due to their lack of arousing potential and texturally simple musical style. Lullabies, and similar music with very carefully managed levels of stimulation and complexity, can be used to help the development of infants with developmental needs [117].

Somewhat related to the use of lullabies, is the way caregivers all over the world also talk to preverbal infants in a special prosodic way characterized by repetitiveness, exaggeration, slowness and simplicity. This is the *parentese* [122], or the way adults speak with babies, which is a kind of infant-directed speech—also called musical speech or child-directed communication—, which tends to be higher in pitch, more rhythmic, slower, and exaggerating pitch contours. It seems to suggest that communication at this early stage of development takes place through musical features [126,127] (see also [133]) such as the use of extended vowels, mellifluous sound, narrow pitch range and repeated pitch contours [1].

A further extension of such soothing sonic environments is the deliberate search for natural soundscapes that provide stimuli in the optimal zone of stimulation. In general, positive evaluation of soundscapes is positively associated with the degree of natural character in the sound environment [134]. Some natural sound environments have been termed *nature’s white noise*, with typical examples such as the blowing of the wind, the sound of the sea, waterfalls, the babbling of a brook, mountain rivers, rain in the woods, and many others. These sounds are valued mostly for their relaxing and calming effect [135], though of course other effects are possible from natural sounds.

Scholars are still in search of causal relationships between the acoustic description of the soundscapes and their possible effects on listeners as biological organisms. Though there is relative unanimity about possible harmful effects of certain auditory characteristics (see [3] for a broad overview), such as high loudness levels, specific spectro-temporal configurations, overmodulation and/or amplification of sounds, etc., there is less unanimity about their possible beneficial effects. These also can be defined negatively in terms of what should be avoided (lower than threshold of pain, above threshold of hearing), or in quite general terms as stimulation within the optimal zone of stimulation. It is even questionable whether this challenge can ever be met, given the overwhelming role of the subjective appraisal and individual learning history of each individual listener. Yet, there are indirect methods of measurement, as used in the study of soundscape dimensions in terms of pleasantness and eventfulness [49,136], or further subdivisions in terms of pleasant/unpleasant, rich (pleasant and eventful), dangerous (unpleasant and eventful), calming (pleasant an uneventful), or boring (unpleasant and uneventful) [33,137]. These reflect the basic dimensions of human mood, as advocated in the seminal work by Russell [48], but other attributions such as calming, protective, hectic, belonging, stability and appropriateness have been proposed as well [46,62]. It is not difficult to translate this to the realm of music, and to select music of different styles and genres and then to ask to describe it in terms of soundscape dimensions. It can be hypothesized, further, that positive descriptions will yield feelings of wellbeing and (see [138] for a broad overview). This suggests two potential directions for future research: first, it would be possible to ask participants to apply scales developed for assessing soundscapes to music. This would provide interesting insight into both how people respond to different pieces of music and into the extent to which the soundscape questionnaires work (and fail) in such an application. Second, by asking people to describe their “musicscape”, their internal response to a piece of music, new aspects of soundscapes may be brought to light that have not been considered in previous research.

## 6. Conclusions

The sound environment and music intersect in several ways: so too do the soundscape and the internal response to listening to music. Music may be part of a sound environment, and may therefore influence the soundscape experienced in response to it; on the other hand, music may take on some aspects of environmental sound, and therefore some of the soundscape response may be experienced alongside the response to the music. At a deeper level, music, spoken language, and the sound environment may all have contributed to our evolution through coping mechanisms, and the cognitive-emotional structures and responses evoked by all three sources of acoustic information may be, to some extent, the same. Our work has attempted both to distinguish and to define the extent of our understanding about how these aspects of external sound and our internal response to it can be explained in terms of relevant areas of science. Future research will allow this to be refined further.
